# Heparanase regulates EMT and cancer stem cell properties in prostate tumors

**DOI:** 10.3389/fonc.2022.918419

**Published:** 2022-07-27

**Authors:** Valentina Masola, Marco Franchi, Gianluigi Zaza, Francesca Mansa Atsina, Giovanni Gambaro, Maurizio Onisto

**Affiliations:** ^1^ Department of Biomedical Sciences, University of Padova, Padova, Italy; ^2^ Department of Life Quality Sciences, University of Bologna, Rimini, Italy; ^3^ Department of Medical and Surgical Sciences, University of Foggia, Foggia, Italy; ^4^ University Hospital of Verona, Verona, Italy; ^5^ Renal Unit, Department of Medicine, University Hospital of Verona, Verona, Italy

**Keywords:** prostate cancer, heparanase, epithelial to mesenchymal transition, cancer stem cells, *in vitro*

## Abstract

Prostate cancer displays a certain phenotypic plasticity that allows for the transition of cells from the epithelial to the mesenchymal state. This process, known as epithelial–mesenchymal transition (EMT), is one of the factors that give the tumor cells greater invasive and migratory capacity with subsequent formation of metastases. In addition, many cancers, including prostate cancer, are derived from a cell population that shows the properties of stem cells. These cells, called cancer stem cells (CSCs) or tumor-initiating cells, not only initiate the tumor process and growth but are also able to mediate metastasis and drug resistance. However, the impact of EMT and CSCs in prostate cancer progression and patient survival is still far from fully understood. Heparanase (HPSE), the sole mammalian endoglycosidase capable of degrading heparan sulfate (HS), is also involved in prostate cancer progression. We had previously proved that HPSE regulates EMT in non-cancerous pathologies. Two prostate cancer cell lines (DU145 and PC3) were silenced and overexpressed for HPSE. Expression of EMT and stemness markers was evaluated. Results showed that the expression of several EMT markers are modified by HPSE expression in both the prostate cancer cell lines analyzed. In the same way, the stemness markers and features are also modulated by HPSE expression. Taken together, the present findings seem to prove a new mechanism of action of HPSE in sustaining prostate cancer growth and diffusion. As for other tumors, these results highlight the importance of HPSE as a potential pharmacological target in prostate cancer treatment.

## Introduction

Of all the existing cancers, prostate cancer is the one that has globally recorded the greatest growth in cases over the last 30 years in both developing and developed countries. Being a male cancer that occurs mainly in old age, the aging of the population is one of the factors that have contributed most to the increase in cases (1). It is estimated that prostate cancer will increase to nearly 2.3 million new cases and 740,000 deaths per year by 2040 simply as a result of population growth and aging (2, 3). The severity of the disease is conferred by the ability of cancer cells to disseminate and metastasize, affecting various organs and tissues—specifically, bone tissue in 84% of cases, distant lymph nodes in 10.6%, the liver in 10.2%, and the thorax in 9.1% (4).

Two phenomena that contribute to the progression and metastasis of this neoplasm are the epithelium–mesenchymal transition process (EMT) and the presence of tumor stem cells. The EMT is a reversible cell-differentiation process during which the morphological and phenotypic conversion of polarized epithelial cells into mesenchymal cells occurs. These cells have a greater migratory capacity, greater resistance to apoptosis, increased expression of mesenchymal markers, and resistance to senescence. This change involves the loss of the junction systems that hold epithelial cells together and the loss of baso-apical polarity and various rearrangements of the cytoskeletal apparatus (5, 6).

The EMT process has also been shown to be present in prostate cancer, and among the various factors that seem to be involved in promoting this change, androgens and estrogens with their related signaling, hypoxia, transforming growth factor beta (TGF-β), and epidermal growth factor (EGF) must be mentioned (7).

These factors promote the activation of the PI3K/AKT and MAPK signaling pathway—thus activating the downstream effectors such as GSK3β and NF-kB, which increase the activity of SNAI-1 and Twist and consequently induce the expression of mesenchymal proteins (8).

Cancer stem cells, according to the American Association for Cancer Research, are described as “a cell within a tumor that possesses the capacity to self-renew and to cause the heterogeneous lineages of cancer cells that comprise the tumor” (9).

Consequently, in line with this definition, CSCs possess both the ability to expand the population of cancer stem cells and, after differentiation, to give rise to other types of neoplastic cells that will make up a large part of the tumor mass. Since non-stem cells in cancer have a limited capacity for proliferation, the only cells with unlimited potential are cancer stem cells, which are thus able to guide the growth and metastatic process (10). For this reason, CSCs that have very long division times, and which are relatively insensitive to drug therapies aimed at targeting rapidly proliferating cells, may be one of the sources of cancer recurrence (11). Over the last few years, experimental evidence has accumulated in favor of the existence of CSC in prostate cancer and its role in tumor and metastatic progression (12). CSCs of the prostate can originate from basal or luminal-type progenitor/stem cells that will develop into tumors with markedly different biological and clinical characteristics in terms of aggressiveness and response to chemotherapy treatments and androgen-deprivation therapy (ADT) (13, 14). Since prostatic CSCs play a fundamental role in ADT resistance, it is currently considered essential to develop new anti-CSC strategies to compensate for treatment failure and disease recurrence (15).

More recently, it has been suggested that the expression of HPSE, the only enzyme capable of degrading the heparan sulfate (HS) chains of HS-proteoglycans (HSPGs), is associated with the characteristics of CSCs in Hodgkin’s lymphoma cells (16) and in myeloma (17).

HS is a highly sulfated linear polysaccharide, attached to the core protein of heparan sulfate proteoglycans (18, 19). HS proteoglycans are ubiquitously found both at the cell surface (i.e., syndecans and glypicans) and in the extracellular matrix (ECM) (18) where they regulate ECM structure and cell–ECM interaction (20, 21). In addition, HS regulates the activity of several molecules (cytokines, growth factors, etc.) (22–25). Currently, heparanase (HPSE) is defined as a multitasking protein capable of performing enzymatic-degradative activity towards HS chains, but which, at the same time, also manifests non-enzymatic activities (26). Through its cutting activity of the side chains of heparane sulfates (HS), it contributes both to the remodeling of the extracellular matrix and to the release and diffusion of various bioactive molecules linked to HS such as growth factors, cytokines, and enzymes. Considering that heparanase is not only produced and secreted by cancer cells but also by endothelial cells and activated immune cells and platelets, it is not surprising that its activity has a strong impact on the tumor microenvironment, thanks to those factors linked to HS, which, once released, promote tumor growth, neo-angiogenesis, and the formation of a metastatic niche (27). As a proof of concept, it has been shown that heparanase overexpression in transgenic mice (Hpa-Tg) makes the tumor microenvironment more conducive to neoplastic development in various experimental models of *in vivo* tumorigenesis (28, 29).

Since prostate cancer also has an increased expression of HPSE (30), we decided to verify whether this increase could be able to regulate EMT and cancer stem cells properties of prostate cancer.

## Methods

### Cell lines

DU145 (ATCC^®^ HTB-81™) and PC3 (ATCC^®^ CRL-1435™) prostate cancer cell lines were cultured in Roswell Park Memorial Institute (RPMI) containing 10% fetal bovine serum and supplemented with 1% penicillin/streptomycin. Cells were maintained in a humidified environment containing 5% CO_2_ at 37°C, and the culture medium was replaced every 2 days.

### Transfection of HPSE overexpressing and shRNA plasmid

In order to stably obtain HPSE-overexpressing DU145 cells, we used a plasmid-coding HPSE ORF purchased from OriGene, and as a negative control, we used the corresponding empty vector.

To stably obtain HPSE-silenced PC3 cells, we used four different shRNAs targeting human heparanase (NM_006665) purchased from OriGene as described earlier (31, 32). As a negative control, we used an shRNA pRS non-effective GFP plasmid (TR30003).

DU145 and PC3 cells were seeded in six-well plates and when they reached 70%–80% of confluence, they were transfected with Lipofectamine 3000 (Invitrogen) according to the manufacturer’s instructions. Forty-eight hours after transfection, DU145 cells overexpressing HPSE were selected with 500 μg/ml G418 (Sigma), and PC3 cells silenced for HPSE were selected with 0.75 μg/ml of puromycin (Sigma). Single clones were isolated and analyzed for HPSE expression. The ones with the highest overexpression/silencing rate were used in the subsequent experiments.

### RNA isolation and real-time qPCR analysis

Total RNA was extracted from cells by Trizol reagent (Invitrogen) according to the manufacturer’s instructions (33). RNA yield and purity were checked using a Nanodrop spectrophotometer (EuroClone), and total RNA from each sample was reverse transcribed into cDNA using Moloney Murine Leukemia Virus Reverse Transcriptase (Sigma-Aldrich). Real-time PCR was performed on a StepOne™ Real-Time PCR System (Thermo Fisher) using SensiFAST SYBR Hi-Rox (Bioline). The comparative Ct method (ΔΔCt) was used to quantify gene expression, and the relative quantification was calculated as 2^−ΔΔCt^. The presence of non-specific amplification products was excluded by melting curve analysis. Statistical analyses on real-time PCR data were performed using the Relative Expression Software Tool (REST) (34). The forward and reverse primer sequences were reported in **Table 1**.

**Table 1:** Primer sequences used for real-time PCR .

Gene	Forward Sequence (5'–3')	Reverse Sequence (5'–3')	Product Length (bp)
GAPDH	ACACCCACTCCTCCACCTTT	TCCACCACCCTGTTGCTGTA	112
HPSE	ATTTGAATGGACGGACTGC	GTTTCTCCTAACCAGACCTTC	136
E-CAD	TTCTGCTGCTCTTGCTGTTT	TGGCTCAAGTCAAAGTCCTG	142
VIM	AAAACACCCTGCAATCTTTCAGA	CACTTTGCGTTCAAGGTCAAGAC	74
α-SMA	GAAGAAGAGGACAGCACTG	TCCCATTCCCACCATCAC	143
TGF-β	CGTGGAGCTGTACCAGAAAT	GATAACCACTCTGGCGAGTC	90
SDC1	GAAGATCAAGATGGCTCTGGG	GTTCTGGAGACGTGGGAATAG	145
SOX2	AGCTACAGCATGATGCAGGA	GGTCATGGAGTTGTACTGCA	126
OCT4	CCTCACTTCACTGCACTGG	CAGGTTTTCTTTCCCTAGCT	164
NANOG	CAGTCTGGACACTGGCTGAA	CTCGCTGATTAGGCTCCAAC	149
CD133	TCAGTGAGAAAGTGGCATCG	GCTTTTCCTATGCCAAACCA	121

### Western blotting and immunofluorescence

Cells were lysed in radioimmunoprecipitation assay buffer (RIPA) buffer composed of 150 mM NaCl, 50 mM TRIS_HCl (pH 8), 0.5% sodium deoxycholate, 0.1% sodium dodecyl sulfate (SDS), and 1% Triton-X with Complete Protease Inhibitor Mixture (Roche Applied Science, Penzberg, Germany). In brief, equal amounts of proteins were treated in reducing sample buffer and denatured for 10 min at 100°C. Protein samples were then resolved in 10% sodium dodecyl sulfate–polyacrylamide gel electrophoresis (SDS–PAGE) and electrotransferred to nitrocellulose membranes. Non-specific binding was blocked for 1 h at room temperature with non-fat milk (5%) in TBST buffer (50 mM Tris–HCl, pH 7.4, 150 mM NaCl and 0.1% Tween 20). Membranes were exposed to primary antibodies GAPDH (sc-47778 Santa Cruz), HPSE (MA1-83806 HP3/17, Thermo Fisher), E-cadherin (E-CAD) (GTX10443 GeneTex), vimentin (VIM) (sc-7557 Santa Cruz), α-SMA (A5228 Sigma), SOX2 (GTX101507 GeneTex), OCT4 (GTX627419 GeneTex), and NANOG (GTX100863 GeneTex), overnight at 4°C and incubated with a secondary peroxidase-conjugated antibody for 1 h at room temperature. The signal was detected with Luminata™ Forte Western HRP Substrate (Millipore) according to the manufacturer’s instructions, and the signal was acquired with Mini HD9 (UVItec, Cambridge, UK). Immunofluorescence cells were fixed in 4% paraformaldehyde for 15 min, permeabilized with 0.2% Triton X-100 in phosphate-buffered saline (PBS) for 5 min, and blocked with 3% bovine serum albumin (BSA) in PBS at room temperature for 30 min. Cells were then incubated overnight at 4°C with the primary antibodies in PBS with 1% BSA and then washed three times for 5 min with PBS before incubation for 1 h at 37°C with the secondary antibody, again in PBS with 1% BSA. Cell nuclei were visualized with a Hoechst 33258. Images were obtained with a confocal LeicaSP5 microscope.

### Colony formation assay

WT and HPSE-silenced/overexpressing prostate cancer cells were seeded in 35-mm culture dishes (1,000 cells per well) and incubated with RPMI supplemented with 10% FBS for 7–10 days (35, 36). The media was renewed every 2 days. The colonies were fixed using paraformaldehyde and stained with 0.1% crystal violet. Cell colony-forming ability was assessed by counting the number of colonies. A colony was defined when the number of cells was more than 50.

### Hanging drop assay

To assess the spheroid-formation capacity and compare the spheroid size of the cells, 500 cells in DMEM-F12 complete medium were placed as drops (20 μl each) into the lid of a Petri dish. The lid was then rapidly re-inverted onto the culture dish that was filled with 10 ml of sterile PBS to prevent evaporation of the drops. The hanging drop cultures were incubated at 37°C in a humidified atmosphere with 5% CO_2_ for 1 week. Pictures of the spheroids inside the drop were taken using a Leica MZ16F stereomicroscope, and their comparative size was obtained measuring the area occupied by the spheres using the software NIH ImageJ.

## Results

### Establishment of HPSE overexpressing/silenced prostate cancer cell lines

**Figure 1:**
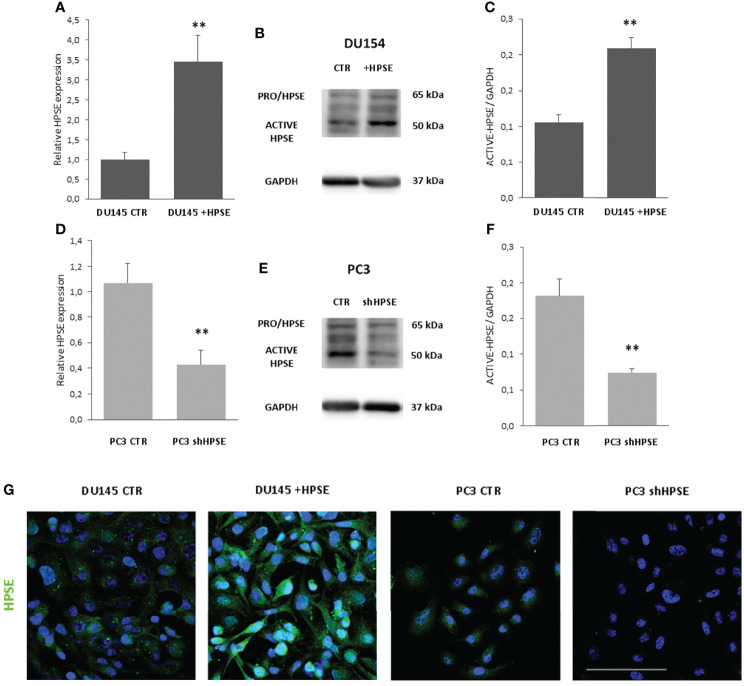
HPSE overexpression and silencing in prostate cancer cells. HPSE gene expression was evaluated by real-time PCR on DU145 **(A)** and PC3 **(D)** cells. Data were normalized to GAPDH expression. Means ± SD (error bars), n=6. **p < 0.001 vs. CTR. WB analysis of HPSE in DU145 **(B)** and PC3 **(E)** and relative quantification **(C, F)**. **(G)** HPSE immunofluorescence in DU145 and PC3 cells (green). Nuclei were counterstained with Hoechst 33342. Scale bar = 100 μm.

In order to investigate the function of HPSE in prostate cancer, we choose to use two prostate cancer cell lines because they display very different morphological aspects related to EMT. DU145 has a more epithelial phenotype, whereas PC3 cells are elongated and spindle-shaped like mesenchymal cells. Starting from this evidence, we decided to overexpress HPSE in DU145 and to silence it in PC3 (**Supplementary Figure S1**). HPSE was overexpressed in DU145 cells and silenced in PC3 cells. Results confirmed a significant HPSE upregulation in DU145 cells both at gene and protein level confirmed by WB and immunofluorescence (**Figure 1**) with respect to DU145 control cells transfected with the empty vector (CTR). CTR cells displayed HPSE expression levels comparable to wt DU145 cells (data not shown). Furthermore, HPSE was silenced by shRNA in PC3 cells by more than 50% both at gene and protein levels (**Figure 1**).

### HPSE regulates EMT in prostate cancer cells

**Figure 2:**
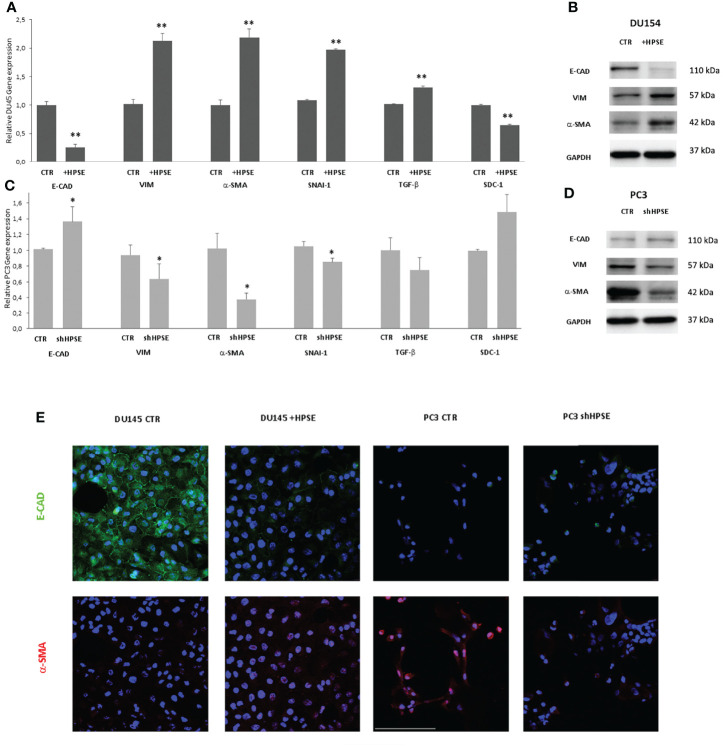
EMT markers expression in prostate cancer cells. E-CAD, VIM, α-SMA, TGF-β, and SDC-1 gene expression was evaluated by real-time PCR in DU145 **(A)** and PC3 **(C)** cells. Data were normalized to GAPDH expression. Means ± SD (error bars), n=6. **p < 0.001, *p<0.05 vs. CTR. E-CAD, VIM, a-SMA, and protein level were quantified by WB in DU145 **(B)** and PC3 **(D)** cells. GAPDH was included as loading control. **(E)** E-CAD (green) and α-SMA (red) immunofluorescence in DU145 and PC3 cells. Nuclei were counterstained with Hoechst 33342. Scale bar = 100 μm.

In order to investigate whether HPSE is able to modulate EMT, the expression levels of epithelial and mesenchymal markers were measured in DU145 and PC3 cells. Gene expression analyses indicated a decrease in epithelial marker E-cadherin in HPSE overexpressing DU145 cells compared with the control. Also observed was a marked increase in the expression levels of vimentin, *α*-SMA, SNAI1, and TGF-β compared with the control (**Figure 2A**). By contrast, in HPSE-silenced PC3 cells, E-cadherin gene expression was increased, and the expression of mesenchymal markers was reduced (**Figure 2C**). The reduction in E-cadherin and the increase in vimentin and a-SMA in HPSE-overexpressing DU145 cells were also confirmed at protein level by WB and by immunofluorescence (**Figures 2B, E**). On the other hand, the reduction in vimentin and a-SMA in HPSE-silenced PC3 cells was also confirmed at protein level (**Figures 2D, E**). These results indicate that HPSE likely promotes EMT in prostate cancer cells.

HS proteoglycans has an important role on prostate epithelium–stroma architecture (34). In particular, syndecans, a family of heparan sulfate proteoglycans that are present on the cell surface, are involved in the control of cell proliferation, apoptosis, and transformation. In prostate cancer, the expression of syndecan-1 in epithelial cells decreases when cells are transformed and acquire invasive properties. This decreased expression is associated with a bad prognosis (37–39).

In addition, HPSE cleaves HS chains on syndecan-1, and a tight relationship between HPSE and syndecan-1 has been documented in tumor and non-tumor models (19, 32, 40, 41).

Results showed that syndecan-1 gene expression was reduced in HPSE-overexpressing DU145 cells, and, in contrast, syndecan-1 expression was increased in HPSE-silenced PC3 cells.

### HPSE regulates stemness features in prostate cancer cells

**Figure 3:**
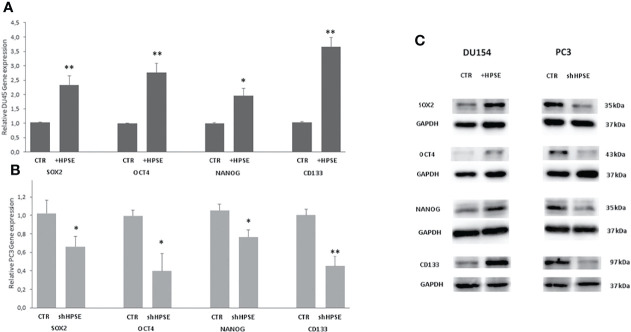
Stemness markers expression in prostate cancer cells. SOX2, OCT4, NANOG, and CD133 gene expression were evaluated by real-time PCR in DU145 **(A)** and PC3 **(B)** cells. Data were normalized to GAPDH expression. Means ± SD (error bars), n=6. **p < 0.001, *p<0.05 vs. **(C)** SOX2, OCT4, and NANOG protein level were quantified by WB in DU145 and PC3 cells. GAPDH was included as loading control.

Numerous studies have shown that the key regulators in maintaining the stemness of embryonic stem cells, including Oct4, Sox2, and Nanog, along with their activation targets, are commonly overexpressed in cancer stem cells in several malignancies (42–44). In addition, a series of molecules, including CD44, CD133, integrin α2β1, ALDH1A1, and Bmi1, involved in the regulation of cancer stem cell self-renewal, metastasis, and drug resistance, have also been confirmed in prostate cancer (45, 46). Results showed that the stemness markers SOX2, OCT4, NANOG, and CD133 were upregulated both at gene and protein level in HPSE-overexpressing DU145 cells compared with the control. By contrast, the same markers were significantly reduced in HPSE-silenced PC3 cells compared with the control (**Figure 3**).

Two important characteristics of cancer stem cells are the capacity to grow starting from a single cell and to form a sphere in an independent anchoring system.

**Figure 4:**
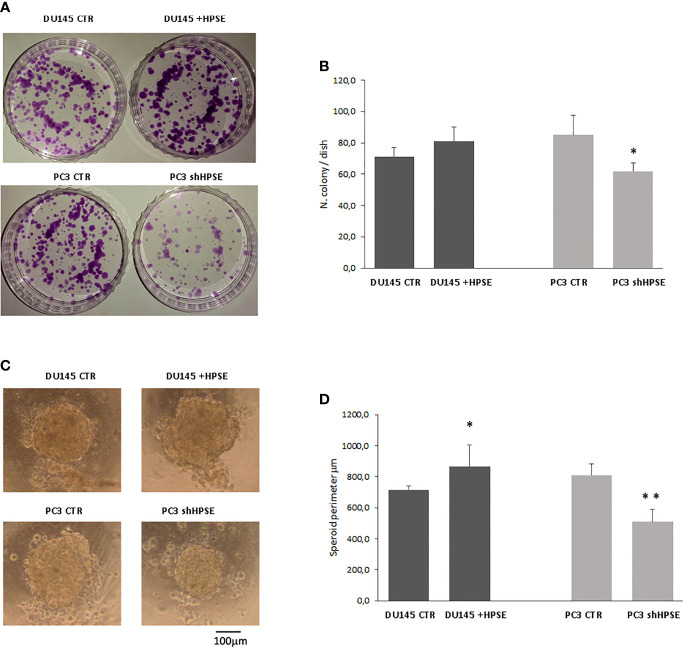
Stemness properties in prostate cancer cells. Representative images of colony **(A)** and sphere **(C)** assays evaluated in DU145 and PC3 cells. **(B, D)** Bars represent the quantification of colony and sphere assay respectively and are expressed as mean ± SD values; n=8. **p < 0.001, *p<0.05 vs. CTR.

Colony formation is the ability of cancer stem cells to form colonies when seeded on cell culture dishes at very low concentrations after limiting dilutions (47). Results showed that HPSE overexpression in DU145 cells increases the number of colonies compared to control cells and that HPSE silencing in PC3 significantly limited the ability to form colonies (**Figures 4A, B**).

To assess the spontaneous sphere formation efficiency of cancer stem cells, we used the hanging drop method (48, 49). Both cell lines were able to form similar circular spheres. HPSE overexpression in DU145 cells increased the sphere perimeter, whereas HPSE silencing in PC3 cells exerted the opposite effects (**Figures 4C, D**).

## Discussion

The malignant growth and progression of tumor disease are supported by key features of cancer cells that have collectively been referred to as “hallmarks of cancer.” To the initial six hallmarks (supporting proliferative signaling, evading growth suppressors, replicative immortality, resistance to cell death, inducing angiogenesis, and activating invasion and metastasis), four other “enabling features” have more recently been added, such as instability and mutation of the genome, tumor-promoting inflammation, deregulated energy metabolism, and escape from immune destruction (50). Since it is now consolidated evidence that all tumors examined to date overexpress HPSE, some authors have proposed that with its multiple roles within the tumor microenvironment, heparanase can regulate each of these distinctive characteristics of cancer and, in turn, highlight the need for therapies aimed at its inhibition (51).

Starting from this evidence, our goal was to evaluate how the overexpression and silencing of heparanase in prostate cancer cells affect stemness characteristics and epithelium–mesenchymal transition (EMT)—two of the classic tumor “hallmarks.”

EMT is thought to be activated in cancer cells, linked to their dissociation from the primary tumor and their intravasation into blood vessels (52). However, the impact of EMT in cancer progression and patient survival is still far from fully understood.

EMT was originally described during morphogenesis and later was observed in several pathological events, including fibrosis and cancer metastasis (53). During EMT, epithelial cells lose adherence junctions and (54) apical–basal polarity and acquire a mesenchymal phenotype with an enhanced motility. In response to various signals (55–58), epithelial cells upregulate a group of transcription factors to orchestrate EMT, and the main ones are SNAI-1 and TWIST. EMT can be considered to be a continuum process (59), and cells with this hybrid phenotype have been referred to as “metastable” (60), reflecting the flexibility of these cells in inducing or reversing the EMT process (61).

Heparanase is the sole mammalian endoglycosidase capable of degrading glycosaminoglycan HS. The enzymatic cleavage of HS by HPSE results in ECM remodeling and releasing these bioactive mediators, producing a rapid tissue response to local or systemic stimuli. These effects profoundly affect multiple pathophysiological processes such as tumor progression, inflammation, and fibrosis (62–65). Since uncontrolled HS degradation results in significant tissue damage, HPSE expression is tightly regulated (19, 66, 67), whereas it is overexpressed in malignant tumors (66, 68).

Prostate cancer is responsible for more gender-specific cancer-related deaths in men than any other cancer (1). Zhou et al. (69) have shown that HPSE overexpression can facilitate tumor invasion and accelerate bone destruction caused by prostate cancer metastasis. Expression of HPSE has also been evaluated in prostate neoplasia; its malignant transformation has been shown to be associated with heparanase-1 increased expression at both mRNA and protein levels (70). These authors have also correlated HPSE expression with the degree of metastasizing tumors and suggested its use as a potential marker for diagnosis of the prostate metastatic process (70).

We and other previous literature support the contention that heparanase is needed for pathological organ fibrosis, and this action is exerted thorough the regulation of EMT. *In vitro* and *in vivo* studies have shown that HPSE regulates renal EMT induced by FGF-2, TGF-beta, and hypoxia (71–74). We have also proved that HPSE regulates high glucose-induced EMT of mesothelial cells (75). In addition, it has been proven that HPSE participates in lung fibrosis by regulating EMT (76), and it probably also has a role in the liver (33).

We induced HPSE overexpression in DU145 cell line and its silencing, by shRNA, in PC3 cell line. The silencing/overexpression rate was confirmed both at gene and protein levels. Results showed that HPSE is an EMT inducer in prostate cancer. Indeed, the epithelial marker E-cadherin was reduced in HPSE-overexpressing DU145 cells and upregulated in HPSE-silenced PC3 cells with respect to control. In contrast, the expression of mesenchymal markers a-SMA and vimentin was increased in HPSE-overexpressing DU45 cells and reduced in HPSE-silenced PC3 cells with respect to control.

Moreover, we have proved that HPSE regulates the expression of TGF-β (one of the principal EMT activators) and the levels of its associated transcription factor SNAI-1. Specifically, TGF-b and SNAI-1 were increased in HPSE-overexpressing DU145 cells and reduced in HPSE-silenced PC3 cells with respect to control.

During malignant transformation depletion of epithelial cell surface, syndecan-1 profoundly alters cell morphology and anchorage-dependent growth: thus, syndecan-1 is necessary in maintaining the epithelial phenotype. TGF-β can induce EMT by activating SNAI-1, which in turn, represses the expression of syndecan-1. A coordinated loss of syndecan-1 and E-cadherin has been documented in many epithelial malignancies compared to their benign counterparts. In the prostate, changes in syndecan-1 expression are linked to EMT (77). It has been described that syndecan-1 expression is lower in PC3 and DU145 prostate cancer cell lines than in normal prostate epithelial cells (78).

Here, we have shown that HPSE expression modulates syndecan-1: HPSE overexpression reduced syndecan-1 in DU145 cells, and HPSE silencing increased syndecan-1 expression in PC3 cell line.

Recently, EMT has been linked to stem cell phenotype (79), since cancer cells with EMT characteristics acquires stem-cell-like features, such as self-renewal and slow proliferation (80, 81). Cancer stem cells acquire more complete EMT molecular characteristics and exhibit more aggressive abilities. Specifically, prostate cancer stem cells display increased EMT markers and increased tumorigenesis, migration, and invasion ability (82). Cancer stem cells have a specific gene signature, and the principal markers are SOX2, OCT4, and NANOG. Additionally, other genes involved in maintaining self-renewal capacity in prostate cancer include CD-133 and CD-44 (83, 84).

Here, we have proved that HPSE expression modulates prostate CSCs. Specifically, the stemness markers SOX2, OCT4, NANOG, and CD133 were upregulated both at gene and protein levels in HPSE-overexpressing DU145 cells and reduced in HPSE-silenced PC3 cells compared with the control. In addition, functional assays confirmed a role of HPSE in prostate cancer stemness: HPSE increases the capacity to grow starting from a single cell and to form a sphere in an independent anchoring system. Future studies could also characterize the potential role of HPSE in self-renewal capacity of prostate CSCs.

Here, we report that the expression of several EMT markers is controlled by HPSE expression in prostate cancer. Moreover, stemness markers and features of CSCs are also modulated by HPSE. Collectively, these results proved an additional mechanism by means of which HPSE can contribute to prostate cancer progression and metastasis, and further studies will be necessary to clarify its potential as a pharmacological goal.

## Data availability statement

The original contributions presented in the study are included in the article/**Supplementary Material**. Further inquiries can be directed to the corresponding authors.

## Author contributions

VM, MO, and GG conceptualized this study. GZ and MF contributed to the design and implementation of the research. VM and FA carried out the experiment. VM and MO wrote the manuscript. All authors have read and agreed to the published version of the manuscript.

## Funding

This work was supported by a grant provided by the University of Padova (BIRD192859/19).

## Conflict of interest

The authors declare that the research was conducted in the absence of any commercial or financial relationships that could be construed as a potential conflict of interest.

The handling editor AP declared a past co-authorship with the authors MF, MO, VM.

## Publisher’s note

All claims expressed in this article are solely those of the authors and do not necessarily represent those of their affiliated organizations, or those of the publisher, the editors and the reviewers. Any product that may be evaluated in this article, or claim that may be made by its manufacturer, is not guaranteed or endorsed by the publisher.

## References

[1] Dy GW Gore JL Forouzanfar MH Naghavi M Fitzmaurice C. Global burden of urologic cancers, 1990-2013. Eur Urol (2017) 71(3):437–46. doi: 10.1016/j.eururo.2016.10.008 28029399

[2] Zhou CK Check DP Lortet-Tieulent J Laversanne M Jemal A Ferlay J. Prostate cancer incidence in 43 populations worldwide: An analysis of time trends overall and by age group. Int J Cancer (2016) 138(6):1388–400. doi: 10.1002/ijc.29894 PMC4712103 26488767

[3] Culp MB Soerjomataram I Efstathiou JA Bray F Jemal A. Recent global patterns in prostate cancer incidence and mortality rates. Eur Urol (2020) 77(1):38–52. doi: 10.1016/j.eururo.2019.08.005 31493960

[4] Gandaglia G Abdollah F Schiffmann J Trudeau V Shariat SF Kim SP. Distribution of metastatic sites in patients with prostate cancer: A population-based analysis. Prostate (2014) 74(2):210–6. doi: 10.1002/pros.22742 24132735

[5] Lamouille S Xu J Derynck R. Molecular mechanisms of epithelial-mesenchymal transition. Nat Rev Mol Cell Biol (2014) 15(3):178–96. doi: 10.1038/nrm3758 PMC4240281 24556840

[6] Kalluri R Weinberg RA. The basics of epithelial-mesenchymal transition. J Clin Invest (2009) 119(6):1420–8. doi: 10.1172/JCI39104 PMC2689101 19487818

[7] Montanari M Rossetti S Cavaliere C D’Aniello C Malzone MG Vanacore D. Epithelial-mesenchymal transition in prostate cancer: an overview. Oncotarget (2017) 8(21):35376–89. doi: 10.18632/oncotarget.15686 PMC5471062 28430640

[8] Di Zazzo E Galasso G Giovannelli P Di Donato M Bilancio A Perillo B. Estrogen receptors in epithelial-mesenchymal transition of prostate cancer. Cancers (Basel) (2019) 11(10):1418. doi: 10.3390/cancers11101418 PMC6826537 31548498

[9] Clarke MF Dick JE Dirks PB Eaves CJ Jamieson CHM Jones DL. Cancer stem cells–perspectives on current status and future directions: AACR workshop on cancer stem cells. Cancer Res (2006) 66(19):9339–44. doi: 10.1158/0008-5472.CAN-06-3126 16990346

[10] Clarke MF Fuller M. Stem cells and cancer: two faces of eve. Cell (2006) 124(6):1111–5. doi: 10.1016/j.cell.2006.03.011 16564000

[11] Dean M Fojo T Bates S. Tumour stem cells and drug resistance. Nat Rev Cancer (2005) 5(4):275–84. doi: 10.1038/nrc1590 15803154

[12] Li JJ Shen MM. Prostate stem cells and cancer stem cells. Cold Spring Harb Perspect Med (2019) 9(6):a030395. doi: 10.1101/cshperspect.a030395 30291148 PMC6546034

[13] Wang ZA Shen MM. Revisiting the concept of cancer stem cells in prostate cancer. Oncogene (2011) 30(11):1261–71. doi: 10.1038/onc.2010.530 21119602

[14] Shen MM Wang X Economides KD Walker D Abate-Shen C. Progenitor cells for the prostate epithelium: roles in development, regeneration, and cancer. Cold Spring Harb Symp Quant Biol (2008) 73:529–38. doi: 10.1101/sqb.2008.73.050 19150960

[15] Seruga B Ocana A Tannock IF. Drug resistance in metastatic castration-resistant prostate cancer. Nat Rev Clin Oncol (2011) 8(1):12–23. doi: 10.1038/nrclinonc.2010.136 20859283

[16] Troschel FM Linsenmaier M Borrmann K Eich HT Götte M Greve B. Heparanase expression is associated with cancer stem cell features and radioresistance in hodgkin’s lymphoma cells. Anticancer Res (2021) 41(7):3299–308. doi: 10.21873/anticanres.15117 34230125

[17] Tripathi K Ramani VC Bandari SK Amin R Brown EE Ritchie JP. Heparanase promotes myeloma stemness and *in vivo* tumorigenesis. Matrix Biol (2020) 88:53–68. doi: 10.1016/j.matbio.2019.11.004 31812535 PMC7261637

[18] Parish CR Freeman C Hulett MD. Heparanase: a key enzyme involved in cell invasion. Biochim Biophys Acta (2001) 1471(3):M99–108. doi: 10.1016/S0304-419X(01)00017-8 11250066

[19] Ramani VC Purushothaman A Stewart MD Thompson CA Vlodavsky I Au JL-S. The heparanase/syndecan-1 axis in cancer: mechanisms and therapies. FEBS J (2013) 280(10):2294–306. doi: 10.1111/febs.12168 PMC3651779 23374281

[20] Theocharis AD Skandalis SS Tzanakakis GN Karamanos NK. Proteoglycans in health and disease: novel roles for proteoglycans in malignancy and their pharmacological targeting. FEBS J (2010) 277(19):3904–23. doi: 10.1111/j.1742-4658.2010.07800.x 20840587

[21] Bishop JR Schuksz M Esko JD. Heparan sulphate proteoglycans fine-tune mammalian physiology. Nature (2007) 446(7139):1030–7. doi: 10.1038/nature05817 17460664

[22] Kato M Wang H Kainulainen V Fitzgerald ML Ledbetter S Ornitz DM. Physiological degradation converts the soluble syndecan-1 ectodomain from an inhibitor to a potent activator of FGF-2. Nat Med (1998) 4(6):691–7. doi: 10.1038/nm0698-691 9623978

[23] Rider CC Mulloy B. Heparin, heparan sulphate and the TGF-β cytokine superfamily. Molecules (2017) 22(5):458–60. doi: 10.3390/molecules22050713 PMC6154108 28468283

[24] Meneghetti MCZ Hughes AJ Rudd TR Nader HB Powell AK Yates EA. Heparan sulfate and heparin interactions with proteins. J R Soc Interface (2015) 12(110):0589. doi: 10.1098/rsif.2015.0589 26289657 PMC4614469

[25] Parish CR. The role of heparan sulphate in inflammation. Nat Rev Immunol (2006) 6(9):633–43. doi: 10.1038/nri1918 16917509

[26] Masola V Bellin G Gambaro G Onisto M. Heparanase: A multitasking protein involved in extracellular matrix (ECM) remodeling and intracellular events. Cells (2018) 7(12):236. doi: 10.3390/cells7120236 PMC6316874 30487472

[27] Masola V Zaza G Gambaro G Franchi M Onisto M. Role of heparanase in tumor progression: Molecular aspects and therapeutic options. Semin Cancer Biol (2020) 62:86–98. doi: 10.1016/j.semcancer.2019.07.014 31348993

[28] Boyango I Barash U Naroditsky I Li J-P Hammond E Ilan N. Heparanase cooperates with ras to drive breast and skin tumorigenesis. Cancer Res (2014) 74(16):4504–14. doi: 10.1158/0008-5472.CAN-13-2962 PMC4134691 24970482

[29] Weissmann M Arvatz G Horowitz N Feld S Naroditsky I Zhang Y. Heparanase-neutralizing antibodies attenuate lymphoma tumor growth and metastasis. Proc Natl Acad Sci U S A (2016) 113(3):704–9. doi: 10.1073/pnas.1519453113 PMC4725485 26729870

[30] Barbosa GO Cervigne NK Carvalho HF Augusto TM. Heparanase 1 involvement in prostate physiopathology. Cell Biol Int (2017) 41(11):1194–202. doi: 10.1002/cbin.10748 28206697

[31] Masola V Gambaro G Tibaldi E Onisto M Abaterusso C Lupo A. Regulation of heparanase by albumin and advanced glycation end products in proximal tubular cells. Biochim Biophys Acta (2011) 1813(8):1475–82. doi: 10.1016/j.bbamcr.2011.05.004 21600934

[32] Masola V Gambaro G Tibaldi E Brunati AM Gastaldello A D’Angelo A. Heparanase and syndecan-1 interplay orchestrates fibroblast growth factor-2-induced epithelial-mesenchymal transition in renal tubular cells. J Biol Chem (2012) 287(2):1478–88. doi: 10.1074/jbc.M111.279836 PMC3256891 22102278

[33] Secchi MF Crescenzi M Masola V Russo FP Floreani A Onisto M. Heparanase and macrophage interplay in the onset of liver fibrosis. Sci Rep (2017) 7(1):14956. doi: 10.1038/s41598-017-14946-0 29097791 PMC5668295

[34] Masola V Bonomini M Onisto M Ferraro PM Arduini A Gambaro G. Biological effects of XyloCore, a glucose sparing PD solution, on mesothelial cells: Focus on mesothelial-mesenchymal transition, inflammation and angiogenesis. Nutrients (2021) 13(7):2282. doi: 10.3390/nu13072282 34209455 PMC8308380

[35] Zhang Y Jin Z Zhou H Ou X Xu Y Li H. Suppression of prostate cancer progression by cancer cell stemness inhibitor napabucasin. Cancer Med (2016) 5(6):1251–8. doi: 10.1002/cam4.675 PMC4924383 26899963

[36] Wang M Ren D Guo W Huang S Wang Z Li Q. N-cadherin promotes epithelial-mesenchymal transition and cancer stem cell-like traits *via* ErbB signaling in prostate cancer cells. Int J Oncol (2016) 48(2):595–606. doi: 10.3892/ijo.2015.3270 26647992

[37] Contreras HR Ledezma RA Vergara J Cifuentes F Barra C Cabello P. The expression of syndecan-1 and -2 is associated with Gleason score and epithelial-mesenchymal transition markers, e-cadherin and beta-catenin, in prostate cancer. Urol Oncol ottobre (2010) 28(5):534–40. doi: 10.1016/j.urolonc.2009.03.018 19450993

[38] Barbosa GO Biancardi MF Carvalho HF. Heparan sulfate fine-tunes stromal-epithelial communication in the prostate gland. Dev Dyn (2021) 250(5):618–28. doi: 10.1002/dvdy.281 33325097

[39] Contreras HR. [Syndecans in the diagnosis and prognosis of prostate cancer]. Rev Med Chil Jan (2010) 138(1):95–101. doi: 10.4067/S0034-98872010000100014 20361158

[40] Rangarajan S Richter JR Richter RP Bandari SK Tripathi K Vlodavsky I. Heparanase-enhanced shedding of syndecan-1 and its role in driving disease pathogenesis and progression. J Histochem Cytochem (2020) 68(12):823–40. doi: 10.1369/0022155420937087 PMC7711244 32623935

[41] Teixeira FCOB Götte M. Involvement of syndecan-1 and heparanase in cancer and inflammation. Adv Exp Med Biol (2020) 1221:97–135. doi: 10.1007/978-3-030-34521-1_4 32274708

[42] Jeter CR Liu B Liu X Chen X Liu C Calhoun-Davis T. NANOG promotes cancer stem cell characteristics and prostate cancer resistance to androgen deprivation. Oncogene (2011) 30(36):3833–45. doi: 10.1038/onc.2011.114 PMC3140601 21499299

[43] Chiou S-H Wang M-L Chou Y-T Chen C-J Hong C-F Hsieh W-J. Coexpression of Oct4 and nanog enhances malignancy in lung adenocarcinoma by inducing cancer stem cell-like properties and epithelial-mesenchymal transdifferentiation. Cancer Res (2010) 70(24):10433–44. doi: 10.1158/0008-5472.CAN-10-2638 21159654

[44] Boumahdi S Driessens G Lapouge G Rorive S Nassar D Le Mercier M. SOX2 controls tumour initiation and cancer stem-cell functions in squamous-cell carcinoma. Nature (2014) 511(7508):246–50. doi: 10.1038/nature13305 24909994

[45] Lin X Farooqi AA Qureshi MZ Romero MA Tabassum S Ismail M. Prostate cancer stem cells: Viewing signaling cascades at a finer resolution. Arch Immunol Ther Exp (Warsz) (2016) 64(3):217–23. doi: 10.1007/s00005-016-0383-0 26846602

[46] Li T Su Y Mei Y Leng Q Leng B Liu Z. ALDH1A1 is a marker for malignant prostate stem cells and predictor of prostate cancer patients’ outcome. Lab Invest (2010) 90(2):234–44. doi: 10.1038/labinvest.2009.127 PMC3552330 20010854

[47] Franken NAP Rodermond HM Stap J Haveman J van Bree C. Clonogenic assay of cells in vitro. Nat Protoc (2006) 1(5):2315–9. doi: 10.1038/nprot.2006.339 17406473

[48] Chen K Li X Li N Dong H Zhang Y Yoshizawa M. Spontaneously formed spheroids from mouse compact bone-derived cells retain highly potent stem cells with enhanced differentiation capability. Stem Cells Int (2019) 2019:8469012. doi: 10.1155/2019/8469012 31191686 PMC6525826

[49] Foty R. A simple hanging drop cell culture protocol for generation of 3D spheroids. J Vis Exp (2011) 51):2720. doi: 10.3791/2720 PMC3197119 21587162

[50] Hanahan D Weinberg RA. Hallmarks of cancer: the next generation. Cell (2011) 144(5):646–74. doi: 10.1016/j.cell.2011.02.013 21376230

[51] Jayatilleke KM Hulett MD. Heparanase and the hallmarks of cancer. J Transl Med (2020) 18(1):453. doi: 10.1186/s12967-020-02624-1 33256730 PMC7706218

[52] Thiery JP Acloque H Huang RYJ Nieto MA. Epithelial-mesenchymal transitions in development and disease. Cell (2009) 139(5):871–90. doi: 10.1016/j.cell.2009.11.007 19945376

[53] Yeung KT Yang J. Epithelial-mesenchymal transition in tumor metastasis. Mol Oncol (2017) 11(1):28–39. doi: 10.1002/1878-0261.12017 28085222 PMC5242415

[54] Nieto MA. Epithelial plasticity: a common theme in embryonic and cancer cells. Science (2013) 342(6159):1234850. doi: 10.1126/science.1234850 24202173

[55] Strutz F Zeisberg M Ziyadeh FN Yang C-Q Kalluri R Müller GA. Role of basic fibroblast growth factor-2 in epithelial-mesenchymal transformation. Kidney Int (2002) 61(5):1714–28. doi: 10.1046/j.1523-1755.2002.00333.x 11967021

[56] Moustakas A Heldin C-H. Mechanisms of TGFβ-induced epithelial-mesenchymal transition. J Clin Med (2016) 5(7):63. doi: 10.3390/jcm5070063 PMC4961994 27367735

[57] Lo H-W Hsu S-C Xia W Cao X Shih J-Y Wei Y. Epidermal growth factor receptor cooperates with signal transducer and activator of transcription 3 to induce epithelial-mesenchymal transition in cancer cells *via* up-regulation of TWIST gene expression. Cancer Res (2007) 67(19):9066–76. doi: 10.1158/0008-5472.CAN-07-0575 PMC2570961 17909010

[58] Gonzalez DM Medici D. Signaling mechanisms of the epithelial-mesenchymal transition. Sci Signal (2014) 7(344):re8. doi: 10.1126/scisignal.2005189 25249658 PMC4372086

[59] Nieto MA Huang RY-J Jackson RA Thiery JP. EMT: 2016. Cell (2016) 166(1):21–45. doi: 10.1016/j.cell.2016.06.028 27368099

[60] Lee JM Dedhar S Kalluri R Thompson EW. The epithelial-mesenchymal transition: new insights in signaling, development, and disease. J Cell Biol (2006) 172(7):973–81. doi: 10.1083/jcb.200601018 PMC2063755 16567498

[61] Rosanò L Spinella F Di Castro V Decandia S Nicotra MR Natali PG. Endothelin-1 is required during epithelial to mesenchymal transition in ovarian cancer progression. Exp Biol Med (Maywood) (2006) 231(6):1128–31. doi: 10.3181/00379727-232-2311128 16741062

[62] Secchi MF Masola V Zaza G Lupo A Gambaro G Onisto M. Recent data concerning heparanase: focus on fibrosis, inflammation and cancer. Biomol Concepts (2015) 6(5–6):415–21. doi: 10.1515/bmc-2015-0021 26552066

[63] Lv Q Zeng J He L. The advancements of heparanase in fibrosis. Int J Mol Epidemiol Genet (2016) 7(4):137–40. PMC5218871 28078057

[64] Sanderson RD Elkin M Rapraeger AC Ilan N Vlodavsky I. Heparanase regulation of cancer, autophagy and inflammation: new mechanisms and targets for therapy. FEBS J (2017) 284(1):42–55. doi: 10.1111/febs.13932 27758044 PMC5226874

[65] Masola V Zaza G Onisto M Lupo A Gambaro G. Impact of heparanase on renal fibrosis. J Transl Med (2015) 13:181. doi: 10.1186/s12967-015-0538-5 26040666 PMC4467599

[66] Meirovitz A Goldberg R Binder A Rubinstein AM Hermano E Elkin M. Heparanase in inflammation and inflammation-associated cancer. FEBS J (2013) 280(10):2307–19. doi: 10.1111/febs.12184 PMC3651782 23398975

[67] Ilan N Elkin M Vlodavsky I. Regulation, function and clinical significance of heparanase in cancer metastasis and angiogenesis. Int J Biochem Cell Biol (2006) 38(12):2018–39. doi: 10.1016/j.biocel.2006.06.004 16901744

[68] Nadir Y Brenner B. Heparanase multiple effects in cancer. Thromb Res (2014) 133 Suppl 2:S90–94. doi: 10.1016/S0049-3848(14)50015-1 24862152

[69] Zhou Y Song B Qin W-J Zhang G Zhang R Luan Q. Heparanase promotes bone destruction and invasiveness in prostate cancer. Cancer Lett (2008) 268(2):252–9. doi: 10.1016/j.canlet.2008.04.008 18487013

[70] Kutsenko OS Kovner AV Mostovich LA Kunin IS Nepomnyashchikh RD Prudnikova TY. Expression of heparanase-1 in prostate gland tumors. Bull Exp Biol Med (2012) 152(3):344–7. doi: 10.1007/s10517-012-1524-z 22803082

[71] Masola V Onisto M Zaza G Lupo A Gambaro G. A new mechanism of action of sulodexide in diabetic nephropathy: inhibits heparanase-1 and prevents FGF-2-induced renal epithelial-mesenchymal transition. J Transl Med (2012) 10:213. doi: 10.1186/1479-5876-10-213 23095131 PMC3562257

[72] Masola V Zaza G Secchi MF Gambaro G Lupo A Onisto M. Heparanase is a key player in renal fibrosis by regulating TGF-β expression and activity. Biochim Biophys Acta (2014) 1843(9):2122–8. doi: 10.1016/j.bbamcr.2014.06.005 24937189

[73] Masola V Zaza G Gambaro G Onisto M Bellin G Vischini G. Heparanase: A potential new factor involved in the renal epithelial mesenchymal transition (EMT) induced by Ischemia/Reperfusion (I/R) injury. PLoS One (2016) 11(7):e0160074. doi: 10.1371/journal.pone.0160074 27467172 PMC4965068

[74] Abassi Z Hamoud S Hassan A Khamaysi I Nativ O Heyman SN. Involvement of heparanase in the pathogenesis of acute kidney injury: nephroprotective effect of PG545. Oncotarget (2017) 8(21):34191–204. doi: 10.18632/oncotarget.16573 PMC5470960 28388547

[75] Masola V Granata S Bellin G Gambaro G Onisto M Rugiu C. Specific heparanase inhibition reverses glucose-induced mesothelial-to-mesenchymal transition. Nephrol Dial Transplant (2017) 32(7):1145–54. doi: 10.1093/ndt/gfw403 28064160

[76] He L Sun F Wang Y Zhu J Fang J Zhang S. HMGB1 exacerbates bronchiolitis obliterans syndrome *via* RAGE/NF-κB/HPSE signaling to enhance latent TGF-β release from ECM. Am J Transl Res (2016) 8(5):1971–84. PMC4891412 27347307

[77] Szatmári T Ötvös R Hjerpe A Dobra K. Syndecan-1 in cancer: Implications for cell signaling, differentiation, and prognostication. Dis Markers (2015) 2015:796052. doi: 10.1155/2015/796052 26420915 PMC4569789

[78] Hu Y Sun H Owens RT Gu Z Wu J Chen YQ. Syndecan-1-dependent suppression of PDK1/Akt/bad signaling by docosahexaenoic acid induces apoptosis in prostate cancer. Neoplasia (2010) 12(10):826–36. doi: 10.1593/neo.10586 PMC2950332 20927321

[79] Mani SA Guo W Liao M-J Eaton EN Ayyanan A Zhou AY. The epithelial-mesenchymal transition generates cells with properties of stem cells. Cell (2008) 133(4):704–15. doi: 10.1016/j.cell.2008.03.027 PMC2728032 18485877

[80] Pirozzi G Tirino V Camerlingo R Franco R La Rocca A Liguori E. Epithelial to mesenchymal transition by TGFβ-1 induction increases stemness characteristics in primary non small cell lung cancer cell line. PLoS One (2011) 6(6):e21548. doi: 10.1371/journal.pone.0021548 21738704 PMC3128060

[81] Bisson I Prowse DM. WNT signaling regulates self-renewal and differentiation of prostate cancer cells with stem cell characteristics. Cell Res (2009) 19(6):683–97. doi: 10.1038/cr.2009.43 19365403

[82] Luo Y Cui X Zhao J Han Y Li M Lin Y. Cells susceptible to epithelial-mesenchymal transition are enriched in stem-like side population cells from prostate cancer. Oncol Rep (2014) 31(2):874–84. doi: 10.3892/or.2013.2905 24316717

[83] Desai B Ma T Zhu J Chellaiah MA. Characterization of the expression of variant and standard CD44 in prostate cancer cells: identification of the possible molecular mechanism of CD44/MMP9 complex formation on the cell surface. J Cell Biochem (2009) 108(1):272–84. doi: 10.1002/jcb.22248 PMC7198262 19582779

[84] Richardson GD Robson CN Lang SH Neal DE Maitland NJ Collins AT. CD133, a novel marker for human prostatic epithelial stem cells. J Cell Sci (2004) 117(Pt 16):3539–45. doi: 10.1242/jcs.01222 15226377

