# Supplementary figures and images for: Heparanase regulates EMT and cancer stem cell properties in prostate tumors
Source: Front Oncol. 2022 Jul 27;12:918419. doi: 10.3389/fonc.2022.918419 (PMC9363836; doi:10.3389/fonc.2022.918419)

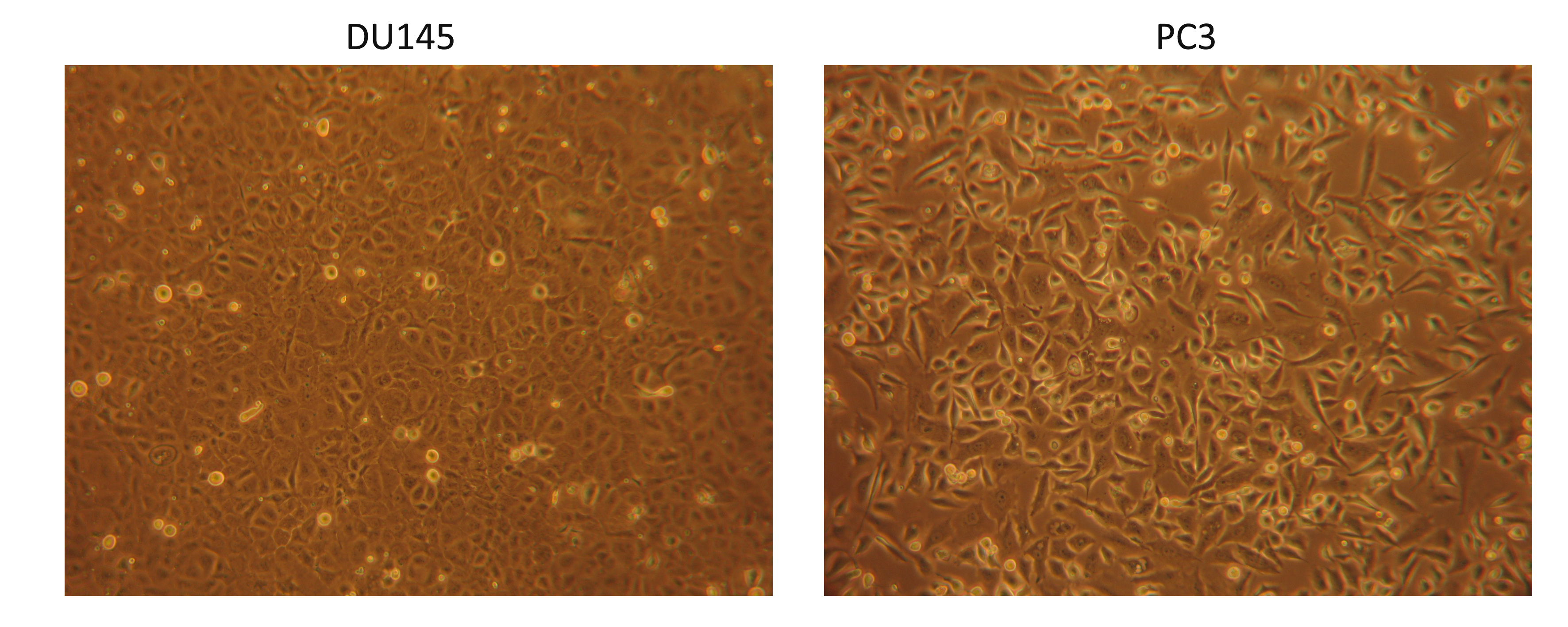

Supplement: Supplementary file 1 [file Image_1.jpeg]
